# False-positive dengue IgM in *Streptobacillus moniliformis* rat-bite fever: the need to look beyond the rapid test—a case report

**DOI:** 10.1128/asmcr.00108-24

**Published:** 2025-05-08

**Authors:** Srishti Chhabra, Yeong Tze Wilnard Tan, Yihui Chen, Jun Yang Tay

**Affiliations:** 1 Department of Medicine, Division of Infectious Diseases, National University Hospital 569029 , Singapore, Singapore; 2 Department of Infectious Diseases, Tan Tock Seng Hospital 663084 , Singapore, Singapore; 3 Department of Laboratory Medicine, Tan Tock Seng Hospital 715102, Singapore, Singapore; Vanderbilt University Medical Center, Nashville, Tennessee, USA

**Keywords:** rat-bite fever, *Streptobacillus moniliformis*, dengue, false positive, case report

## Abstract

**Background:**

This case report explores a unique instance of rat-bite fever (RBF) caused by *Streptobacillus moniliformis*, with an unexpected false-positive result on dengue serology, making the initial diagnosis challenging.

**Case Summary:**

A 47-year-old male presented with acute onset of fever, sore throat, and migratory polyarthritis in a dengue-endemic region. His laboratory investigations revealed a normal white blood cell count, thrombocytopenia, a mildly raised C-reactive protein, and positive dengue IgM serology. He did not report any high-risk sexual exposures or recent diarrheal illness. Initial differential diagnoses included viral arthritis, disseminated gonococcal infection, and reactive arthritis. On the fourth day of admission, his blood cultures returned positive for *Streptobacillus moniliformis,* and the same organism was isolated through 16S rRNA sequencing of his right knee synovial fluid. He denied any history of rat bites and was unaware of any rodent contact. Treatment was commenced with intravenous ceftriaxone, later transitioning to benzylpenicillin and oral amoxicillin, resulting in significant symptom resolution and full recovery of joint function.

**Conclusion:**

This case highlights the diagnostic challenge posed by cross-reactive dengue serology in RBF, emphasizing the need to avoid relying solely on IgM positivity for diagnosing dengue fever. Although comparatively less common, clinicians should be aware of non-specific clinical presentations of RBF, particularly in dengue-endemic areas, and that molecular diagnostic techniques may aid in correct identification and management.

## INTRODUCTION

Rat-bite fever (RBF) is an infrequent, yet potentially fatal illness caused by the pathogens *Streptobacillus moniliformis*, prevalent in the United States and Europe, and *Spirillum minus*, found primarily in Asia, especially Japan (1, 2). Despite these regional tendencies, there are documented instances of *Streptobacillus moniliformis*-related RBF in countries such as India, China, Thailand, and Singapore, suggesting that the geographical boundaries of these pathogens may overlap (3–5). Human transmission occurs through rat bites, contact with rodent secretions, or consumption of contaminated food or water (2). Other animals, including ferrets, squirrels, and domestic pets like dogs and cats, have also been identified as carriers (6).

Diagnosing RBF is challenging due to its non-specific clinical presentation, which includes symptoms such as fever, myalgia, headache, and arthralgia. These symptoms can mimic viral syndromes and may occur without an overt history of rodent exposure (7, 8). The diagnosis is further complicated by the fastidious nature of *Streptobacillus moniliformis*, which can delay laboratory confirmation (6). In high-income dengue-endemic regions, initial diagnostic efforts frequently include rapid dengue serology in patients presenting with viral-like symptoms. Further diagnostic workup may not be pursued once a patient with a viral syndrome has a positive NS-1 antigen or IgM.

In this context, our report presents a unique case where a patient with RBF exhibited a positive dengue serology (IgM), complicating his diagnostic pathway. The dengue IgM positivity, combined with a negative NS-1 antigen and IgG, prompted further investigation for potential cross-reactive alphaviruses, flaviviruses, and other non-dengue etiologies.

## CASE PRESENTATION

A 47-year-old Bangladeshi gentleman, without significant medical history, presented with a 4-day history of fever, sore throat, and acute pain in his right knee, bilateral ankles, and left elbow. He denied any chronic joint or back pain. He had traveled to Bangladesh 6 months earlier, and his last sexual exposure was with his regular partner there. He reported no dysuria, penile discharge, rash, or known animal contact. He lived in a flat with five housemates and worked in a door manufacturing factory.

On examination, he was febrile, with a temperature of 39.2°C, a heart rate of 112 beats per minute, and blood pressure of 114/65 mmHg. Physical examination revealed mild pharyngeal injection and a moderate right knee effusion, displaying warmth and restricted movement due to pain, but no erythema. Similarly, warmth and tenderness were noted in both ankles and the left elbow without significant effusion. The rest of his joints were quiescent without any deformities. Heart, lung, and abdominal examinations were unremarkable. No rash was noted.

Initial laboratory findings showed a white cell count of 7.4 × 10^9^/L (normal range [NR] 4.0–9.6 × 10^9^/L), mild lymphopenia (1.0 × 10^9^/L, NR 1.1–3.0 × 10^9^/L), and thrombocytopenia (platelet count 126 × 10^9^/L, NR 150–360 × 10^9^/L). Kidney and liver functions were normal. C-reactive protein (CRP) was elevated at 68.7 mg/L (NR 0.0–5.0 mg/L). SD Bioline Dengue Duo, a rapid immunochromatographic test, returned positive for dengue IgM but was negative for non-structural protein (NS1) antigen and dengue IgG. Radiographs of the right knee and ankles were normal. The initial impression was a viral infection-associated arthritis syndrome. An alternative consideration was disseminated gonococcal infection, given the presence of oligoarthritis and tenosynovitis of bilateral ankles and left elbow, although the patient had neither a rash nor a typical exposure history. The managing team sent off blood cultures, dengue and chikungunya virus serum polymerase chain reaction (PCR), and urinary PCR for chlamydia and gonorrhea.

**Fig 1:**
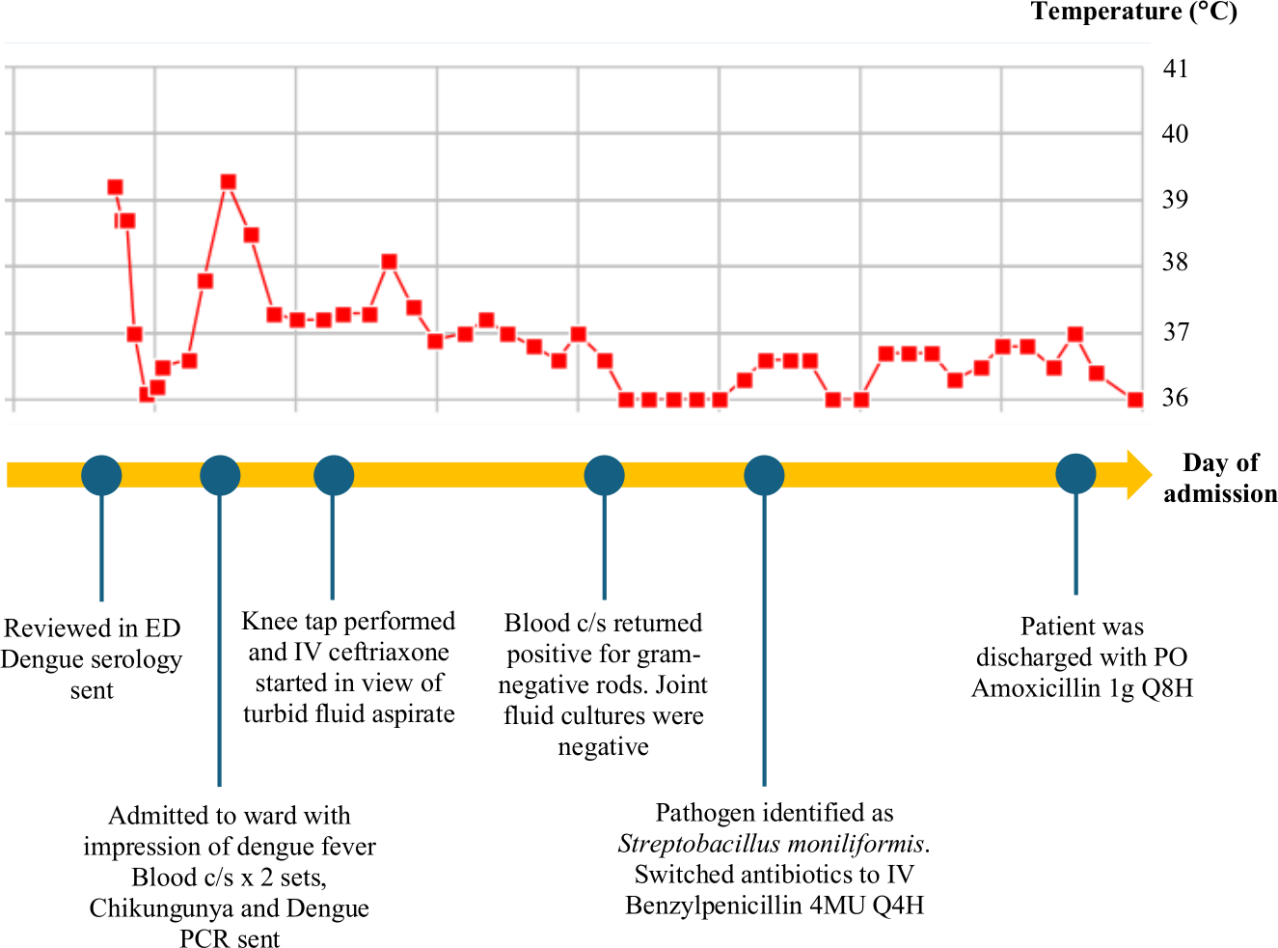
Timeline of events, with each column representing 1 day of admission. The patient’s temperature is shown, with each row indicating a 1°C rise in temperature.

**Fig 2:**
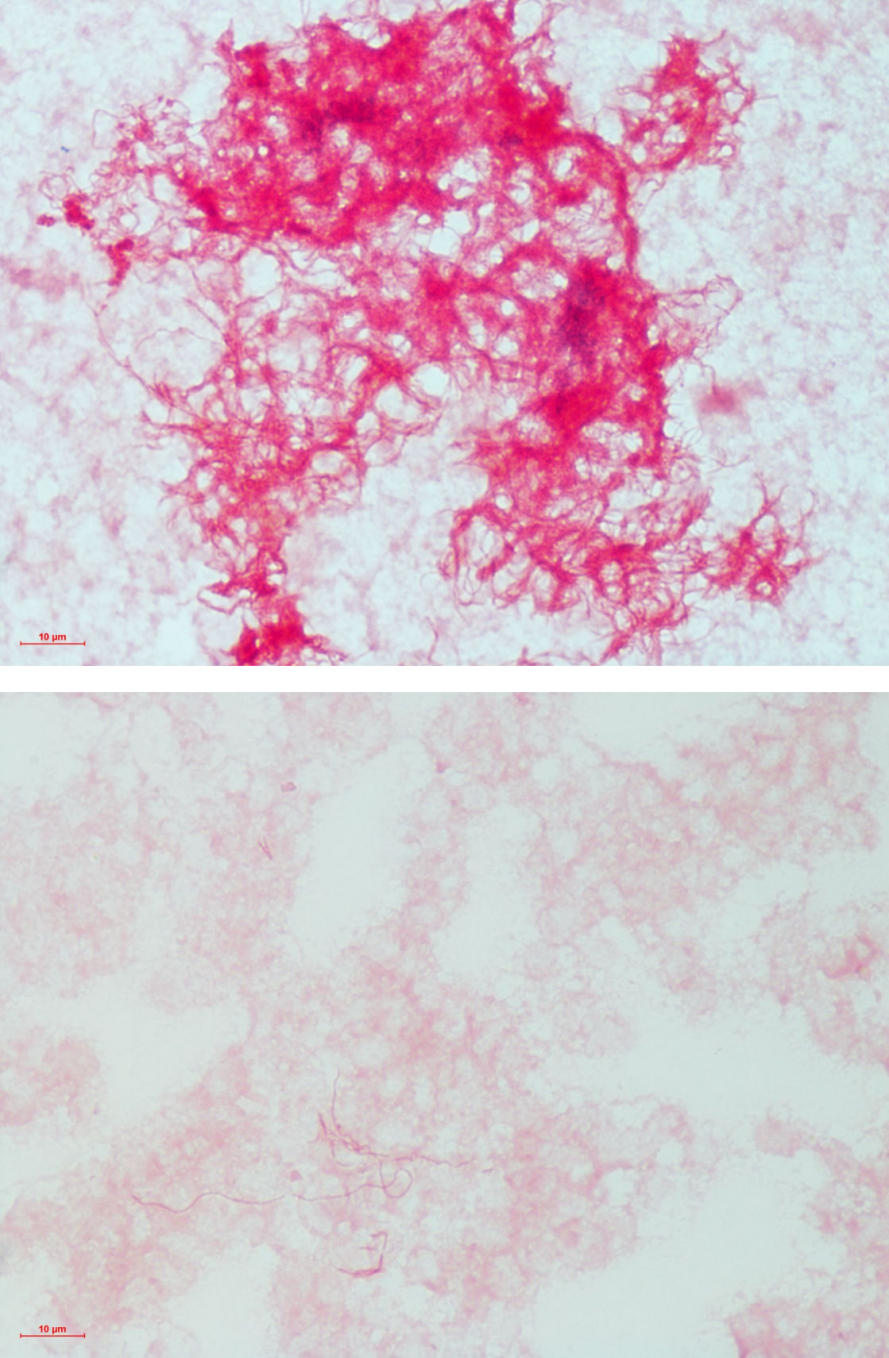
Gram stain of *Streptobacillus moniliformis*. A pleomorphic, clumped, filamentous gram-negative rod seen on anaerobic (top) and aerobic (bottom) blood culture bottles. Images are shown at 1,000× magnification.

On day 2 of admission, he remained febrile and continued to report migratory joint pains, which now involved his left knee and right shoulder (Fig. 1). A right knee aspiration yielded turbid yellow synovial fluid with a white cell count of 58,800 cells/µL, of which 91% were neutrophils. The patient was started on IV Ceftriaxone 2 g daily, after which his fevers abated and joint pains improved. On day 4 of admission, his blood cultures returned positive for a pleomorphic, filamentous gram-negative rod (Fig. 2), which was identified to species level as *Streptobacillus moniliformis* using matrix-assisted laser desorption ionization-time of flight mass spectrometry from the sub-cultured colonies with a score of 2.47 in the aerobic bottle and 2.09 in the anaerobic bottle (Bruker Daltonics). His knee aspirate cultures did not show any bacterial growth using conventional methods, but *Streptobacillus moniliformis* was detected by 16S rRNA gene sequencing. His dengue and chikungunya virus serum PCR and chlamydia and gonorrhea urinary PCR returned negative.

The patient was transitioned to IV benzylpenicillin 4 million units Q4 hourly and later to oral amoxicillin 1 g Q8 hourly, for a total of 2 weeks. He was reviewed in the clinic 2 weeks after discharge, demonstrating full recovery and restored function in his right knee.

## DISCUSSION

*Streptobacillus moniliformis* is a filamentous, pleomorphic, non-motile gram-negative organism found in aggregates or chains (6). It is fastidious, requiring microaerophilic growth conditions. Sodium polyanethol sulfonate, an anticoagulant used in blood culture media, inhibits its growth in concentrations as low as 0.0125%, and prolonged incubation of up to 7 days may be required to isolate the organism. Special broths enriched with 20% blood, serum, or ascitic fluid may be required for optimal growth (6).

The symptoms of RBF and dengue fever have significant overlap—in both conditions, patients can present with fever, myalgia, arthralgia, nausea, vomiting, and headache (6, 9). Rashes may occur in both, although the rash associated with dengue fever is typically macular or maculopapular and becomes diffusely erythematous (10), while that of RBF is often maculopapular, which can develop into purpuric vesicles or pustules (11). The typical biochemical features described in RBF include leukocytosis with neutrophilia and a high CRP (8, 12), while characteristic findings in dengue fever include leukopenia and thrombocytopenia (13). In our patient’s case, however, his white cell count was within normal limits, CRP was only mildly elevated, and he was thrombocytopenic. In Singapore, where dengue fever is endemic, it is common practice to perform a rapid dengue test on patients who present with fever and thrombocytopenia. Further diagnostic workup for viral-associated fevers may not be pursued when a patient has a positive dengue NS1 antigen or IgM, which may have resulted in a missed diagnosis and adverse outcomes for this patient.

While the dengue IgM has a sensitivity of 71%–80%, its specificity is considerably lower, ranging from 46% to 90% (14). There have been reports of dengue IgM cross-reactivity with other viruses, such as Zika virus, West Nile virus, tick-borne encephalitis virus, and SARS-CoV-2 (15, 16). Therefore, we screened for other cross-reactive viruses such as Chikungunya. Dengue IgM cross-reactivity has also been described with autoimmune conditions such as systemic lupus erythematosus (17). To our knowledge, this is the first case report describing a false-positive dengue IgM in a patient with *Streptobacillus moniliformis* RBF.

RBF-associated arthritis is typically polyarticular, involving both small and large joints, with a predilection for the knee (8). A rheumatoid pattern with symmetrical involvement of the small joints of the hand has been described in several cases (18, 19). While synovial fluid analyses usually yield a high leukocyte count with neutrophilic predominance, fluid cultures may be negative. Molecular methods such as 16S rRNA sequencing can aid in pathogen identification from synovial fluid samples (8). Wang et al. (20) proposed clinically distinct patterns of joint involvement in RBF, one being reactive arthritis, and the other being septic arthritis. Patients are less likely to have fevers, positive blood cultures, or a rash in the latter. Our patient did not have the typical vasculitic rash-associated with RBF, and his joint involvement was in keeping with tenosynovitis with left knee monoarthritis.

*Streptobacillus* RBF with septic arthritis has good outcomes with appropriate treatment (8, 20). The optimal treatment of RBF septic arthritis has yet to be established, but current recommendations suggest IV penicillin. Cephalosporins, tetracyclines, and streptomycin have been used successfully in patients with penicillin allergy (6). Unlike most cases of septic arthritis, only 30% of *Streptobacillus* arthritis require joint washout or surgery. This is especially important when there is prosthetic joint involvement (8). There is no clear consensus on the duration of treatment for *Streptobacillus* septic arthritis. Treatment with antibiotic durations of 10 days–6 weeks has had good outcomes. Most reports suggest treatment for 4 weeks (8, 20), but our patient showed complete clinical response to 2 weeks of therapy.

There were several diagnostic challenges in this case: the absence of rodent exposure, biochemical features suggestive of a viral etiology, and a false-positive dengue serology. Due to the fastidious nature of the organism, microbiological identification only occurred 4 days into his admission. This case underscores the critical need for integrating rapid test results with comprehensive biochemical and clinical evaluations rather than relying exclusively on IgM findings. Sole reliance on IgM positivity could have led to inappropriate treatments and potential adverse outcomes. Clinicians should also be aware of the clinical presentation of RBF and that the history of rat exposure may not always be present. Where there are suggestive clinical features, RBF should be considered in cases of culture-negative septic arthritis, and the laboratory should be consulted to optimize culture conditions of clinical samples and advice on adjunctive molecular testing.
